# Germline mutations among Polish patients with acute myeloid leukemia

**DOI:** 10.1186/s13053-021-00200-2

**Published:** 2021-10-12

**Authors:** Aneta Bąk, Katarzyna Skonieczka, Anna Jaśkowiec, Anna Junkiert-Czarnecka, Marta Heise, Maria Pilarska-Deltow, Stanisław Potoczek, Maria Czyżewska, Olga Haus

**Affiliations:** 1 Department of Clinical Genetics, Collegium Medicum, Nicolaus Copernicus University, Bydgoszcz Toruń, Poland; 2 Department of Hematology, Blood Neoplasms and Bone Marrow Transplantation, Medical University, Wrocław, Poland; 3 Department of Hematology, Municipal Hospital, Toruń, Poland

**Keywords:** Acute myeloid leukemia, AML, germline mutations, *CEBPA*, *DDX41*, *ETV6*, *TERT*, *GATA2*, *IDH2*

## Abstract

**Background:**

A small but important proportion of patients (4–10 %) with AML have germline mutations. They can cause the development of AML at an earlier age, confer a higher risk of relapse or predispose to secondary leukemias, including therapy-related leukemias. The analysis of germline mutations in a patient and his/her family is also critical for the selection of suitable family donors if the patient is a candidate for hematopoietic stem cell transplantation (HSCT).

**Methods:**

103 unrelated consecutive patients with *de novo* AML were enrolled in the study. Control group consisted of 103 persons from the general population. We performed NGS sequencing of bone marrow cells and buccal swabs DNA of six genes: *CEBPA, DDX41, ETV6, TERT, GATA2*, and *IDH2* to detect germline pathogenic mutations.

**Results:**

In the investigated group, 49 variants were detected in six genes. 26 of them were somatic and 23 germline. Germline variants were detected in all six tested genes. Eight pathogenic germline mutations were detected in 7 AML patients, in three genes: *CEBPA, ETV6*, and *IDH2*. One patient had two pathogenic germinal mutations, one in *ETV6* and one in *CEBPA* gene. We identified one novel pathogenic germline mutation in *CEBPA* gene. The difference in frequency of all pathogenic germline mutations between the tested (7.77 %) and control groups (0.97 %) was statistically significant (*p* = 0.046). In the tested group, the median age at AML diagnosis was 11 years lower in patients with pathogenic germline mutations than in patients without them (*p* = 0.028).

**Conclusions:**

We showed higher frequency of *CEBPA, ETV6*, and *IDH2* germline mutations in AML patients than in control group, which confirms the role of these mutations in the development of AML. We also showed that the median age at the onset of AML in patients with pathogenic germline mutations is significantly lower than in patients without them.

**Supplementary Information:**

The online version contains supplementary material available at 10.1186/s13053-021-00200-2.

## Background

AML (acute myeloid leukemia) is one of the most common types of leukemia in adults. AML and MDS (myelodysplastic syndromes) exist along a continuous disease spectrum starting with early-stage MDS, which may progress to advanced MDS, AML, resistant or cured AML. The disease is characterized by an overproduction of immature blood cells in the bone marrow (BM) and a lack of mature, healthy blood cells in the peripheral blood. This causes anemia and an increased risk for bleeding and infection. Moreover, there is a dysplasia in one to three main myeloblastic (erythro-, thrombo-, and granulopoietic) cell lines in BM. It was recognized that not only somatic mutations in neoplastic tissue but also germline mutations may influence disease development, progress, and prognosis. This is supported by the fact that the myeloid neoplasms with genetic predisposition represent a new category in the revised 2016 World Health Organization classification. The *RUNX1* (Runt-related transcription factor 1), *CEBPA* (CCAAT enhancer binding protein), *DDX41* (DEAD-box helicase 41), *ETV6* (ETS translocation variant gene 6), *GATA2* (GATA binding protein 2), *TERC* (Telomerase RNA component) and *TERT* (Telomerase reverse transcriptase) genes are important regulators of hematopoiesis and are frequently involved in the pathomechanism of leukemogenesis [1–3]. The importance of methylation disturbances is also emphasized during the development of AML, causing general hypomethylation of the genome and selective hypermethylation of suppressor genes. Methylation disorders are associated with translocations, rearrangements, and mutations of the *IDH1* (Isocitrate dehydrogenase (NADP(+)1), *IDH2* (Isocitrate dehydrogenase (NADP(+)2), *TET2* (Tet methylcytosine dioxygenase 2) and *DNMT3A* (DNA (cytosine-5-)-methyltransferase 3 alpha) genes leading to changes in their expression. However, despite the growing knowledge of germline predisposition to myeloid malignancies, it is not fully explained yet [3, 4].

We performed NGS sequencing of six genes: *CEBPA, DDX41, ETV6, TERT, GATA2*, and *IDH2* in the aim to detect germline pathogenic mutations in Polish patients with *de novo* AML.

AML associated with *CEBPA* or *DDX41* mutations occurs without distinct clinical symptoms or antecedent hematological condition [2]. *CEBPA* gene encodes a transcription factor (TF) involved in the regulation of myelopoiesis. Two types of mutations may appear: an N-terminal frame-shift or overproduction mutation and a C-terminal mutation disrupting the DNA binding. Many AMLs with *CEBPA* mutations simultaneously carry 2 mutations (*CEBPA*dm) in *trans* position (on 2 different alleles), whereas single heterozygous mutations (*CEBPA*sm) occur less frequently. Clinically important *CEBPA*dm present in about 7 % of AML patients contain both, N-terminal and C-terminal mutations on the separate allele each. This mutational status is a favorable prognostic factor [5–8].

*DDX41* encodes an RNA helicase, a protein involved in various processes of RNA metabolism, from the transcription to the degradation of RNA, including pre-mRNA splicing, mRNA export, ribosome biogenesis, translation initiation, and gene expression in organelles. However, *DDX41* role in hematopoiesis and leukemogenesis remains unknown. Moreover, the prevalence and penetrance of *DDX41* mutations are unclear [9, 10].

*ETV6* gene encodes a main hematopoietic TF which is a part of a large, ETS family (E26 transformation-specific) composed of 28 TFs. The ETV6 protein plays a crucial role in embryonic development and hematopoietic regulation. The most frequent clinical feature associated with the germline mutations of *ETV6* gene in the context of hematologic malignancy are younger age at the disease onset, platelet dysfunction, and bleeding disorders associated with an elevated risk of MDS/AML. [2, 11].

Mutations of *TERT* which appear frequently (2–19 %) in bone marrow failure syndromes are associated with an elevated risk of MDS/AML. *TERT* encodes the catalytic subunit of telomerase which catalyses the addition of TTAGGG telomeric repeat sequences at the ends of chromosomes in order to stabilize telomere length and achieve cell immortality. The abnormal reactivation of telomerase complex occurs in approximately 90 % of human tumors, and is considered a crucial element for cancer genesis and progression [12–15].

*GATA2* mutations cause the development of familial MDS and often occur in the setting of cytopenias and rare immunological syndromes. The *GATA2* gene encodes a TF which is expressed in hematopoietic stem cells. This protein contains two zinc finger domains that promote protein-protein and protein-DNA communication. GATA2 participates in the formation of early blood and lymphatic vessels [2, 16, 17].

The importance of methylation disturbances is also emphasized during the development of AML, causing general hypomethylation of the genome and selective hypermethylation of suppressor genes. *IDH2* protein, encoded by *IDH2* gene, plays a key role in the process of DNA methylation/demethylation. *IDH2* mutations result in a hypermethylation phenotype, disrupt *TET2* gene function, and impair hematopoietic differentiation. Mutations in *IDH2* have been first reported in glioblastoma multiforme, next in acute myeloid leukemia, and other malignancies such as breast invasive ductal carcinoma, colon adenocarcinoma, lung adenocarcinoma, and oligodendroglioma. Approximately 20 % of AML patients harbor a mutation in the isocitrate dehydrogenase (*IDH*) genes, *IDH1* or *IDH2* [18–21].

The importance of germline predisposition to myeloid malignancies is more and more emphasized due to its clinical significance; necessity of genetic counseling for family members and influence on hematopoietic stem cell donor selection. Assessment of the presence of germline mutations, particularly in younger patients or those with a positive family history, is important for the optimal care of patients [22].

## Materials and methods

DNA samples stored at the Department of Clinical Genetics, Collegium Medicum in Bydgoszcz, Nicolaus Copernicus University in Torun, Poland (CM NCU) were used for the study. A total of 103 consecutive patients (54 men and 49 women) diagnosed with *de novo* AML, according to World Health Organization criteria, regardless of age at AML diagnosis and family history of cancer were involved in the study. None of the three most common in Polish population mutations in the *BRCA1* gene were detected in the study group. The median age at diagnosis was 56 years (men – 53 years, women – 58 years, range 18–89). The pedigrees were made on the basis of the questionnaires. 39.80 % (41/103) of patients reported at least one first- or second-degree relative with cancer. In 33 families (32,04 %) lung, breast, ovary, stomach or colon cancer mainly occurred, in 3 families (2.91 %) Hodgkin’s lymphoma, in one family (0,97 %) 2 cases of AML and in 4 families (3.88 %) other leukemia types. Most investigated patients had M4 (31 patients), M1 (19), and M2 (18) AML cytomorphologic subtypes, according to the French-American-British (FAB) classification. 12 patients had M5, 5 - M3, 2 - M6, 2 - M7, and 1 - M0 subtypes. For thirteen patients, no FAB data were available.

The control group consisted of 103 volunteers out of the general population who on a questionnaire basis had no malignancies at the time of material collection, and originated from families without a history of cancer. Control group persons were matched by age and sex with patients from the investigated group.

Informed consent was obtained from all patients and control healthy persons. The study was approved by the Ethics Committee of the CM NCU.

DNA from 85 PB (peripheral blood) cell samples and 18 BM cell samples, collected at diagnosis of AML, was used for molecular testing. In the control group, the mutations were searched for in DNA from peripheral blood. Genomic DNA was extracted from leukocytes by QIAmp® DNA Mini Kit (QIAGEN) using standard procedures. In mutation-positive patients, the constitutional character of a mutation was verified by analysis of DNA from BS (buccal swabs), collected at AML diagnosis, extracted by Swab-Extract DNA Purification Kit (EURx) using standard procedures.

For the next-generation sequencing (NGS) reaction, the molecular inversion probes (MIPs) designed using the MIPGEN program were used. Procedures for the preparation of probes, hybridization reaction, complete ligation and amplification were based on the methods developed by Yoon et al. 2015 [23, 24]. Sequencing was performed on a MiSeq sequencer analyzer, in paired-end (PE) technology, 2 × 250nt, using Illumina’s v2 kit, according to the manufacturer’s protocol.

Mutation-positive cases were confirmed by sequencing analysis using ABI PRISM 3130 (Applied Biosystems). For Sanger sequencing, exons were amplified by PCR (PCR profiles, primers sequence available upon request). Primers were designed using the Primer3 tool (http://bioinfo.ut.ee/primer3-0.4.0). Sequencing reaction was conducted on PCR product with BigDye Terminator v3.1 Cycle Sequencing Kit (Applied Biosystems), according to the manufacturer’s procedure, on the coding parts of the genes with parts of introns adjacent to 5’ and 3’ ends of all tested exons.

Pathogenicity of identified mutations was evaluated by the VarSome Clinical database. A variant was described as pathogenic if VarSome Clinical showed its pathogenicity or likely pathogenicity. Mutational analysis was performed on NCBI reference sequences: NM_004364.4, NM_032638.5, NM_001987.5, NM_002168.4, NM_016222.4, and NM_198253.3 for *CEBPA, GATA2, ETV6, IDH2, DDX41*, and *TERT*, respectively.

The karyotypes of heparinized BM cells of each patient were assessed at disease diagnosis, using classical GTG-banding and fluorescence in situ hybridization (FISH) techniques.

Statistical analysis included a comparison of the prevalence of variant alleles in the studied and control groups, calculation of odds ratios (ORs) from two-by-two tables, and calculation of statistical significance of differences between various tested groups using the Chi-square test. The Mann-Whitney U test was used to compare the median age in the groups of patients with and without pathogenic germline mutations. The normal distribution was verified using the Kolmogorov-Smirnov test. Statistical analysis was performed using the IBM SPSS 26 statistical package.

## Results

We detected 49 types of variants in six genes (*CEBPA, DDX41, ETV6, TERT, GATA2, IDH2*). Among variants identified in the study were missense, frameshift, duplication, silent, and intronic variants. 26 types of somatic variants were detected: 5 pathogenic, 6 likely pathogenic, 5 of uncertain significance (VUS), and 10 benign. We detected also 23 types of germline variants: 2 pathogenic, 2 likely pathogenic, 1 VUS, and 18 benign (Tables 1 and 2).

**Table 1:** Somatic mutations in *CEBPA, GATA2, ETV6, DDX41, TERT* and *IDH2* found in patients with AML

Gene	Mutation	Amino acid change		Exon/ Intron	Prediction by VarSome	Variant ID	Carriers *n* = 103	Frequency in Europe (%) GnomAD
***CEBPA***	c.179dupC	p.Ser61ValfsTer47		Ex1	pathogenic	COSM18632	1	–
	c.287_311del	p.Gly96AlafsTer56		Ex1	likely pathogenic	COSV57200088	1	–
	c.402G > A	p.Ala134=		Ex1	benign	rs752254340 COSV57196176	1	0.0
	c.536T > C	p.Phe179Ser		Ex1	VUS	–	1	–
	c.573 C > T	p.His191=		Ex1	benign	rs192240793 COSV57200682	1	0.9
	c.937_939dupAAG	p.Lys313dup		Ex1	likely pathogenic	–	1	–
***GATA2***	c.919 C > T	p.Arg307Trp		Ex5	likely pathogenic	COSM306052	1	–
	c.959G > A	p.Arg320His		Ex5	likely pathogenic	rs121964985 COSM249854	1	0.1
	c.1085G > A	p.Arg362Gln		Ex6	likely pathogenic	rs867160952 COSM87004	1	–
***ETV6***	c.463 + 49G > A	NA		Int4	benign	rs17210957	8	–
	c.1090G > T	p.Glu364Ter		Ex6	pathogenic	–	1	–
***DDX41***	c.409 A > G	p.Thr137Ala	Ex5		VUS	–	16	–
***TERT***	c.835G > A	p.Ala279Thr		Ex2	benign	rs61748181 COSM3760906	2	3.6
	c.915G > A	p.Ala305=		Ex2	benign	rs2736098 COSM3760904	15	23.5
	c.1573 + 39G > C	NA		Int2	benign	rs79662648	2	–
	c.1574-16G > C	NA		Int2	benign	rs79698601	1	0.6
	c.2007_2017del	p.Ala670ArgfsTer32		Ex5	pathogenic	–	7	–
	c.2018_2019insTT	p.Gly674SerfsTer41		Ex5	pathogenic	–	7	–
	c.2020G > A	p.Gly674Ser		Ex5	VUS	rs773813809 COSM6919371	7	0.0
	c.2023 C > T	p.Leu675Phe		Ex5	VUS	–	7	–
	c.3184G > A	p.Ala1062Thr		Ex15	benign	rs35719940 COSM5019566	1	2.2
	c.3324G > A	p.Pro1108=		Ex16	benign	rs35033501 COSM5020092	2	2.9
***IDH2***	c.327G > A	p.Val109=		Ex3	benign	rs150943639 COSM6867404	1	0.4
	c.404 C > A	p.Pro135His		Ex4	likely pathogenic	–	1	–
	c.515G > T	p.Arg172Met		Ex4	pathogenic	rs121913503 COSM33732	2	–
	c.709 C > A	p.Gln237Lys		Ex6	VUS	–	6	–

VUS - variant of uncertain significance, Variant ID - variant identifier, NA - no amino acid change, (–) - no data

**Table 2:** Germline mutations in *CEBPA, GATA2, ETV6, DDX41, TERT*, and *IDH2* found in patients with AML

Gene	Mutation	Amino acid change	Exon/ Intron	Prediction by VarSome	Variant ID	Carriers *n* = 103	Frequency in Europe (%) GnomAD
***CEBPA***	c.337_344del	p.Ala113ArgfsTer54	Ex1	likely pathogenic	–	1	–
	c.590_591insACCCGC	p.Pro198_Ala199dup	Ex1	likely pathogenic	COSM7448245	4	–
	c.690G > T	p.Thr230=	Ex1	benign	rs34529039 COSV57196771	14	16.00
***GATA2***	c.15 C > G	p.Pro5=	Ex3	benign	rs1573858 COSM3749812	21	66.40
	c.481 C > G	p.Pro161Ala	Ex4	benign	rs34799090 COSM5762885	1	0.30
	c.490G > A	p.Ala164Thr	Ex4	benign	rs2335052 COSM445531	23	18.30
	c.564G > C	p.Thr188=	Ex4	benign	rs34870876	19	5.90
	c.1018-19 C > T	NA	Int5	benign	rs11708606	27	19.60
	c.1233G > A	p.Ala411=	Ex7	benign	rs34172218 COSM5019736	1	2.40
***ETV6***	c.258G > A	p.Thr86=	Ex3	benign	rs11611479 COSM3752958	12	10.90
	c.380G > A	p.Arg127Gln	Ex4	benign	rs140357643	1	–
	c.1075 C > T	p.Arg359Ter	Ex6	pathogenic	rs141938078 COSM3456975	1	–
***DDX41***	c.936-23 C > G	NA	Int9	benign	rs335439	56	99.90
	c.1200 C > T	p.Arg400=	Ex11	benign	rs335438 COSM3761138	39	47.10
	c.1302 + 67_1303-67insAG	NA	Int12	VUS	–	1	–
	c.1399 + 46T > C	NA	Int13	benign	–	64	–
	c.1732 + 46 A > G	NA	Int16	benign	–	52	–
***TERT***	c.1770-24 C > T	NA	Int3	benign	rs13167280	15	12.90
	c.1950 + 10 C > T	NA	Int4	benign	rs33948291	1	1.90
	c.3039 C > T	p.His1013=	Ex14	benign	rs33954691 COSM5019111	7	9.70
***IDH2***	c.419G > A	p.Arg140Gln	Ex4	pathogenic	rs121913502 COSM41590	2	0.01
	c.535-40G > A	NA	Int4	benign	rs142033117	2	0.80
	c.996 C > T	p.Ser332=	Ex8	benign	rs61737003 COSM4128570	1	2.60

VUS - variant of uncertain significance, Variant ID - variant identifier, NA - no amino acid change, (–) - no data

Germline variants were detected in each of the six tested genes, but the pathogenic germline mutations were detected only in three genes: *CEBPA, ETV6*, and *IDH2*. We have detected 4 types of mutations in these genes: c.337_344del and c.590_591insACCCGC in *CEBPA*, c.1075 C > T in *ETV6*, and c.419G > A in *IDH2.* Overall, eight pathogenic germline mutations were detected in 7/103 of AML patients, one patient had two pathogenic germline mutations. In the *CEBPA* gene were detected two pathogenic mutations: c.337_344del in one patient and c.590_591insACCCGC in four patients. In one of four patients, c.590_591insACCCGC mutation coexisted with the pathogenic c.1075 C > T mutation in the *ETV6* gene. In the *IDH2* gene c.419G > A mutation occurred in two patients. These mutations were present in DNA not only from BM and/or PB, but also from BS of patients, which confirmed their constitutional character.

In the control group, we detected one pathogenic germline mutation (c.590_591insACCCGC) in the *CEBPA* gene. This mutation was detected in a 27-year-old healthy woman with no family history of cancer. No other variants (somatic or germline) were detected in the tested genes in this woman.

The difference in frequency of all germline mutations between the tested (7.77 %) and control groups (0.97 %) was statistically significant (*p* = 0.046), and the odds ratio was 8.59; 95 % CI: 1.054–69.975 (Table 3). Additionally, in one patient without pathogenic germline mutation, a germline intronic variant of uncertain significance (c.1302 + 67_1303-67insAG) in *DDX41* gene was detected.

**Table 3:** A correlation between pathogenic germline mutations and risk of AML in analyzed patients

Gene	Mutations	Number of pathogenic germline mutations				OR	95 % CI	*p*-value
		***Tested group n = 103*** **[n/%]**		***Control group n = 103*** **[n/%]**				
***CEBPA***	c.337_344del	1	0.97 %	0	0.00 %	3.03	0.122–75.235	*p* = 0.499
	590_591insACCCGC	4	3,88 %	1	0.97 %	4.12	0.453–37.520	*p* = 0.209
***ETV6***	c.1075 C > T	1	0.97 %	0	0.00 %	3.03	0.122–75.235	*p* = 0.499
***IDH2***	c.419G > A	2	1.94 %	0	0.00 %	5.10	0.241-107.521	*p* = 0.295
**All mutations**		8	7.77 %	1	0.97 %	8.59	1.054–69.975	***p = 0.046***

We also analyzed a correlation between pathogenic germline mutations and karyotype of bone marrow cells at AML diagnosis. In the study group, 39 patients had chromosome aberrations, 38 had normal karyotypes, and in 26 the results of cytogenetic diagnostics were not available. 6 patients with germline mutations had normal karyotypes, and one had an aberrant karyotype (Table 4).

**Table 4:** Clinical data of patients with pathogenic germline mutations and co-occurring mutations

Patient laboratory number	Sex/age	AML classification (FAB)	Karyotype	Family history of cancer	Nucleotide change					
					***IDH2***	***CEBPA***	***GATA2***	***ETV6***	***TERT***	***DDX41***
10	M/44	M5	NK	ND	c.419G > A* c.709 C > A	–	c.1018-19 C > T	–	c.1770-24 C > T c.3184G > A	c.936-23 C > G c.1200 C > T
13	K/47	M1	NK	CLL60	–	c.590_591insACCCGC*	–	c.1075 C > A*	–	c.936-23 C > G c.1200 C > T
33	K/37	M6	NK	ND	–	c.337_344del*	–	c.258G > A	c.915G > A	c.409 A > G c.936-23 C > G c.1200 C > T
43	M/20	M1	NK	ND	–	c.590_591insACCCGC*	c.490G > A	c.463 + 49G > A	–	c.409 A > G c.936-23 C > G c.1200 C > T
47	M/51	M4	NK	Pr72, Ut70	–	c.590_591insACCCGC*	–	c.258G > A	–	c.936-23 C > G c.1200 C > T
83	K/49	M4	t(9;11)	Br50	–	c.590_591insACCCGC*	c.15 C > G c.490G > A	–	–	c.936-23 C > G
97	M/48	M1	NK	Bn62	c.419G > A*	c.690G > T	c.15 C > G	–	–	c.936-23 C > G

CLL - chronic lymphocytic leukemia, Pr - prostate cancer, Ut - uterine cancer, Br - breast cancer, Bn - brain cancer, FAB - French-American-British classification, (*) - germline mutation, NK - normal karyotype, ND - no data, (–) - no mutation

We found that 21/103 patients (20.4 %) harbored a somatic and/or germline mutation in *CEBPA* gene. 18 patients had a single mutation, 2 patients two mutations (one somatic and one germline), and 1 patient had three mutations (two somatic and one germline). The location and combination pattern of all the detected *CEBPA* mutations are presented in Table 5.

**Table 5:** Summary of *CEBPA* mutations detected in 21 acute myeloid leukemia patients

Mutation status	Mutation 1			Mutation 2 (Mutation 3)			No. of patients
	N-terminal AA 1-120	Middle AA 121–277	C-terminal AA 278–358	N-terminal AA 1-120	Middle AA 121–277	C-terminal AA 278–358	
*CEBPA*-single	p.Ala113ArgfsTer54*						1
		p.Ala134=					1
		p.His191=					1
		p.Pro198_Ala199dup*					4
		p.Thr230=*					11
*CEBPA*-double	p.Gly96AlafsTer56				p.Thr230=*	(p.Lys313dup)	1
	p.Ser61ValfsTer47				p.Thr230=*		1
		p.Phe179Ser			p.Thr230=*		1

(*) – germline mutation

**Fig. 1:**
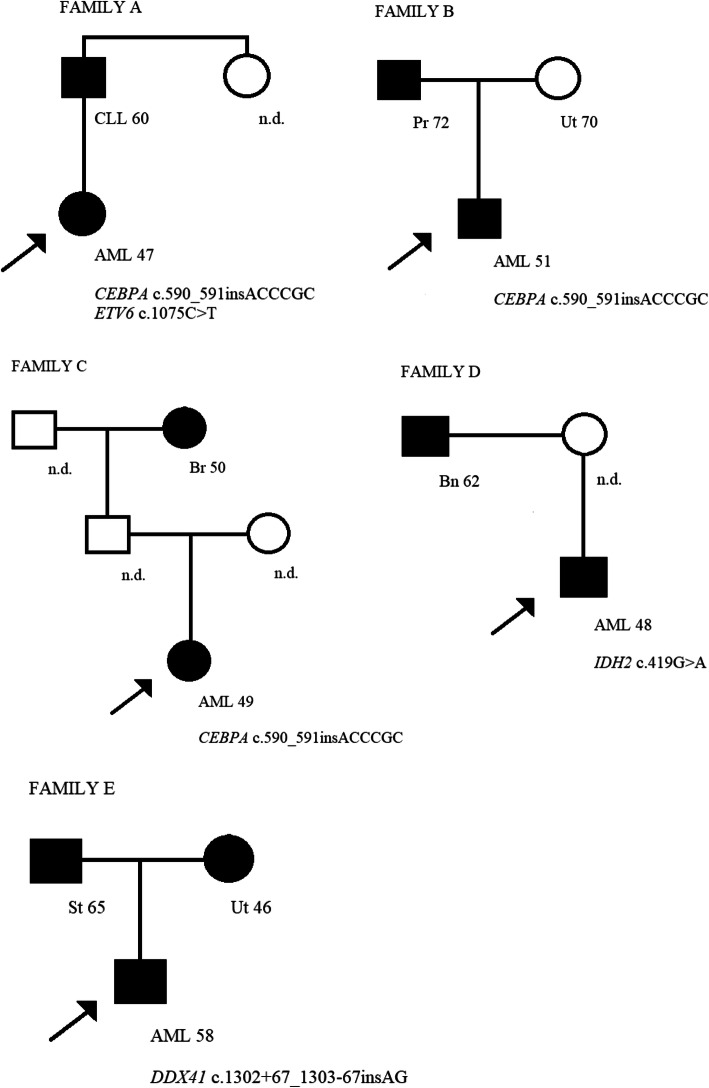
The pedigrees of families with pathogenic germline mutations carriers (**A-D**) and germline variant of uncertain significance carrier (**E**). In all pedigrees, probands are indicated by arrows. The type of cancer and the age at disease diagnosis are described under the filled black square/circle. AML - acute myeloid leukemia, CLL - chronic lymphocytic leukemia, Pr - prostate cancer, Ut - uterine cancer, St - stomach cancer, Br -breast cancer, Bn - brain cancer, n.d. - no data

Single pathogenic germline mutation in *CEBPA* gene was found in 5 out of 103 AML patients (4.9 %), in two patients with M1-AML, two with M4-AML, and one with M6-AML. The c.590_591insACCCGC mutation in *CEBPA* occurred in four unrelated patients (age at diagnosis 20, 47, 49, and 51 years), while c.337_344del mutation occurred in one patient (age at diagnosis 37 years) (Tables 2 and 3). In the family of the patient with AML diagnosed at the age of 20, no other cancer was present. In one patient with M1-AML (age at diagnosis 47 years) two pathogenic germline mutations, c.590_591insACCCGC in *CEBPA* and c.1075 C > A in *ETV6* (the only *ETV6* germline mutation found in analyzed group), occurred. In his family, chronic lymphocytic leukemia (age at diagnosis 60 years) in second-degree relative appeared. In the family of the patient with AML diagnosed at the age of 49, breast cancer (age at diagnosis 50 years) in a second-degree relative occurred. In the family of the patient with AML diagnosed at the age 51, prostate cancer (age at diagnosis 72 years) and uterine cancer (age at diagnosis 70 years) in two first degree-relatives occurred (Fig. 1). In the family of the patient with AML diagnosed at the age of 37, and c.337_344del mutation, no other cancer was present.

The c.419G > A mutation in *IDH2* occurred in two unrelated patients (age at diagnosis 44 and 48 years) with M5-AML and M1-AML, respectively (Table 4). In the family of the patient with AML diagnosed at the age of 44, no other cancer was present. In the family of the patient with AML diagnosed at the age of 48, a brain tumor in the first-degree relative occurred (age at diagnosis 62 years) (Fig. 1).

The c.1302 + 67_1303-67insAG germline intronic variant of uncertain significance in *DDX41* gene occurred in a patient (age at diagnosis 58 years) with M5-AML. Stomach cancer (age at diagnosis 65 years) and uterine cancer (age at diagnosis 46 years) in two first degree-relatives occurred in his family (Fig. 1).

The median age at AML diagnosis was 11 years lower in patients with germline pathogenic mutations (47 years, range 20–51) than in patients without them (58 years, range 18–89; *p* = 0.028).

## Discussion

We tested *CEBPA, GATA2, ETV6, IDH2, DDX41*, and *TERT* genes, to detect germline pathogenic mutations in AML patients. We found germline variants in each of the tested genes, but only 8 of them were pathogenic – 5 in *CEBPA*, 1 in *ETV6*, and 2 in *IDH2* genes. In the *CEBPA* gene were detected: c.337_344del mutation in one patient and c.590_591insACCCGC mutation in four patients, in the *ETV6* gene c.1075 C > T mutation in one patient, and in the *IDH2* gene c.419G > A mutation in two patients. The frequency of all pathogenic germline mutations was 7.77 % in tested group and 0.97 % in control group (*p* = 0.046).

We identified one novel pathogenic germline mutation, c.337_344del, in *CEBPA* gene. This mutation creates a stop codon within the TAD2 domain and results in a truncated protein. In our study, this mutation was disclosed with 0.97 % (1 patient) frequency in the study group, which was not statistically significantly different (NS) from its frequency in control group (no patient with this mutation). Moreover, in the *CEBPA* gene we identified a known pathogenic germline mutation, c.590_591insACCCGC, present with 3.88 % frequency in the study, and 0.97 % in the control group. The difference was not statistically significant, however the odds ratio was 4.12; 95 % CI: 0.453–37.520. According to the latest knowledge, the prevalence of this mutation in the European population has not yet been evaluated (https://varsome.com).

We also found a germline nonsense mutation c.1075 C > T in *ETV6* gene, which creates a stop codon within the ETS domain and results in a truncated protein, without a DNA-binding function. This mutation was disclosed in one female patient (0.97 %) in tested group and in no person in control group (NS). Chronic lymphocytic leukemia (age at diagnosis 60 years) in second-degree relative occurred in patient’s family. *ETV6* mutation coexisted with the c.590_591insACCCGC *CEBPA* mutation in one patient. The coexistence of two germline pathogenic mutations also was reported by Tsaousis et al. in hereditary breast cancer [25]. Moriyama et al. identified the same as in our patient *ETV6* mutation in all investigated females from an acute lymphoblastic leukemia (ALL) family of European descent. The mother and 2 of her 3 daughters developed ALL in the childhood, at the ages of 9, 3, and 2 years, respectively. All 3 ALL cases were of B-lineage, although with various molecular subtypes. Mild congenital thrombocytopenia was noted in mother and one daughter, the second daughter was diagnosed with Turner syndrome and mild intellectual disability, and the third was diagnosed with a learning disability. The enlarged family history did not reveal other hematologic malignancies. Interestingly, the mutation was also present in the healthy daughter, suggesting incomplete penetrance. However, given her young age of 11 it is still possible that she has been at risk of ALL [26].

Mutations in the *IDH1* and *IDH2* genes are well described in lower-grade gliomas (grade II and III astrocytomas and oligodendrogliomas) and secondary glioblastomas, where they have an incidence of more than 70 %. Mutations and polymorphisms in these genes, predominantly somatic, are reported in 5–15 % of AML patients [27–29]. In AML, nearly all *IDH2* mutations cause a single amino acid substitution, Arg172 to one of four different residues - Lys, Met, Gly, and Trp, and Arg140 to either Gln or Trp [29, 30]. Following the discoveries in glioma and AML, mutations in *IDH2* gene were found in multiple types of human tumors, including thyroid carcinomas, cartilaginous tumors, and intrahepatic cholangiocarcinoma [31]. *IDH2* mutations give rise to protein with newly acquired and distinct enzyme activity, which can catalyze NADPH-dependent reduction of α-KG to 2-hydroxyglutarate (2HG). Accumulation of this putative oncogenic metabolite has been observed in malignant gliomas and may be related to the pathogenesis of malignant brain tumors. Increased cellular 2HG levels contribute to epigenetic mechanisms of pathogenesis by inhibiting α-KG-dependent enzymes that are important for normal DNA methylation [32, 33].

A germline missense mutation, c.419G > A, in the *IDH2* gene was detected in two patients in our study. According to GnomAD, this mutation occurs with a frequency of 0.01 % in the European population, as well as in the African and East Asian populations. The prevalence of this mutation was almost two-fold higher in relation to the control group, but this difference was not statistically significant (*p* = 0.295). In the family of one of two patients with c.419G > A mutation, a brain tumor occurred, at 48 years of age. Kranendijk et al. detected heterozygous germline mutations in *IDH2* that alter enzyme residue Arg140 in patients with D-2-hydroxyglutaric aciduria (D-2-HGA). In 15 unrelated patients they detected a known heterozygous c.419G > A (p.Arg140Gln) mutation, the same as in our patients, and a novel heterozygous mutation c.418 C > G (p.Arg140Gly) [34, 35]. Molenaar et al. described a germline *IDH2* mutation, c.782G > A (p.Arg261His), in a patient with AML, and another germline mutation, c.1304 C > T (p.Thr435Met), in two unrelated patients with MDS (RCMD - Refractory Cytopenia with Multilineage Dysplasia) [36].

In our study, one germline intronic variant (c.1302 + 67_1303-67insAG) in the *DDX41* gene was detected, which was identified as VUS in VarSome. This variant occurred in a patient with AML diagnosed at 58 years. Stomach cancer and uterine cancer were diagnosed in two of the first degree-relatives of the patient. So far, this variant has not been described (https://varsome.com).

The results of our study suggest the need to increase the study group as well as to carry out the family studies to determine a heritability of a variant/mutation among cancer-positive family members.

In our study, we were interested only in germline mutations. They may be the best distinguished from somatic mutations by sequencing of a nonhematopoietic tissue, such as fibroblasts grown from a skin biopsy. However, a disadvantage of this approach is that a skin biopsy would be an additional invasive procedure in AML patients and that fibroblasts require several weeks of culture. Thus, we decided on testing buccal swabs. They were taken with due care and caution to avoid contamination with hematological material. Two mutations found in buccal swabs in our patients were confirmed as the germline ones in some other publications [24, 36]. The nature of other mutations found by us (c.337_344del, c.590_591insACCCGC, and c.1302 + 67_1303-67insAG) has not yet been confirmed elsewhere.

In the present study, we found no correlation between pathogenic germline mutations and karyotype of bone marrow cells at AML diagnosis.

## Conclusions

Familial AML predisposition syndromes are rare inherited disorders characterized by significantly elevated risk of AML development. Although several disorders with germline predisposition have been included in the revised 2016 WHO classification of myeloid neoplasms and acute leukemia as “myeloid neoplasms with germline predisposition”, screening for known germline mutations in the background of these syndromes is not a part of the routine diagnostic algorithms. However, if a germline mutation is detected, this should be always taken into account in prophylactic and therapeutic decisions (WHO 2016) [1, 2]. Given the molecular heterogeneity of AML, a better understanding of mutational classes and their involvement in AML pathogenesis could improve risk stratification of patients for more effective and targeted therapy [37]. Identification of the affected families with inherited AML is of critical importance as they not only provide unique models to study the molecular pathogenesis of these diseases, but identification of a germline mutation may have immediate clinical implications as regards the pre-neoplastic monitoring of family members with this mutation. In addition, identification of germline mutations will make it possible to invent prophylactic and early diagnostic issues against germline mutations-driven AML. It is believed that familial AML is still underdiagnosed and its frequency is higher than currently reported. Further studies are necessary to assess the prevalence of germline mutations in the larger AML patient cohort and to establish their prognostic significance.

## Supplementary information

**Additional file 1**

## Data Availability

All information about data and materials will be available on request after prior contact with me at my e-mail address: aneta.bak@cm.umk.pl.

## Abbreviations

AML: Acute myeloid leukemia
HSCT: Hematopoietic stem cells transplantation
DNA: Deoxyribonucleic acid
*CEBPA*: CCAAT/Enhancer-Binding Protein-A
*DDX41*: DEAD/H-box helicase
*ETV6*: EST Variant Transcription Factor 6
*TERT*: Telomerase reverse transcriptase
*GATA2*: GATA bining protein 2
*IDH2*: Isocitrate dehydrogenase (NADP(+))2
NGS: Next generation sequencing
*RUNX1*: Runt-related transcription factor 1
*TERC*: Telomerase RNA component
*IDH1*: Isocitrate dehydrogenase (NADP(+))1
*DNMT3A*: DNA methyltransferase 3 alpha
TF: transcription factor
RNA: Ribonucleic acid
EST: E26 transformation specific
MDS: Myelodysplastic syndromes
*TET2*: Tet methylcytosine dioxygenase 2
CM NCU: Nicolaus Copernicus University in Torun, Poland
FAB: French-American-British classification
PB: Peripheral bloom
BM: Bone marrow
BS: Buccal swabs
MIP: Molecular inversion probe
PE: Paired-end
PCR: Polymerase chain reaction
FISH: Fluorescence in situ hybridization
OR: Odds ratio
VUS: Uncertain significance variant
NS: Not statistically significantly different
ALL: Acute lymphoblastic leukemia
RCMD: Refractory cytopenia with multilineage dysplasia
WHO: World Health Organization
ID: Identifier
NA: No amino acid change
CLL: Chronic lymphocytic leukemia
Pr: Prostate cancer
Ut: Uterine cancer
Br: Breast cancer
Bn: Brain cancer
St: Stomach cancer
NK: Normal karyotype
ND: No data
