# Supplementary material for: Germline mutations among Polish patients with acute myeloid leukemia
Source: Hered Cancer Clin Pract. 2021 Oct 12;19:42. doi: 10.1186/s13053-021-00200-2 (PMC8507332; doi:10.1186/s13053-021-00200-2)
Supplement: Supplementary file 1 — Additional file 1 [file 13053_2021_200_MOESM1_ESM.docx]

FAMILY NAME, FIRST NAME……………………………………………………….…………………..….ID……………………….………………………

DIAGNOSIS…..…………………………………………………………………………………………………………………………………………………………

| Relatives  Born in (year) | Family name, first name | Primary localization of a neoplasm (involved organ) | Age at the diagnosis | If passed away – at which age |
| --- | --- | --- | --- | --- |
| Brothers  1.  2.  3.  4. |  |  |  |  |
| Sisters  1.  2.  3.  4. |  |  |  |  |
| Sons  1.  2.  3.  4. |  |  |  |  |
| Daughters  1.  2.  3.  4. |  |  |  |  |
| Grandchildren |  |  |  |  |
| FATHER |  |  |  |  |
| Father’s brothers  1.  2.  3.  4. |  |  |  |  |
| Father’s sisters  1.  2.  3.  4. |  |  |  |  |
| Father’s father |  |  |  |  |
| Father’s mother |  |  |  |  |
| MOTHER |  |  |  |  |
| Mother’s brothers  1.  2.  3.  4. |  |  |  |  |
| Mother’s sisters  1.  2.  3.  4. |  |  |  |  |
| Mother’s father |  |  |  |  |
| Mother’s mother |  |  |  |  |

……………………………………………………

Date and signature

*The original questionnaire was written in Polish. Here we show an English version of it.
